# The effects of maternal care on the developmental transcriptome and metatranscriptome of a wild bee

**DOI:** 10.1038/s42003-023-05275-2

**Published:** 2023-09-14

**Authors:** Katherine D. Chau, Mariam Shamekh, Jesse Huisken, Sandra M. Rehan

**Affiliations:** Department of Biology, York University, Toronto, Canada

**Keywords:** Social evolution, Behavioural ecology

## Abstract

Maternal care acts as a strong environmental stimulus that can induce phenotypic plasticity in animals and may also alter their microbial communities through development. Here, we characterize the developmental metatranscriptome of the small carpenter bee, *Ceratina calcarata*, across developmental stages and in the presence or absence of mothers. Maternal care had the most influence during early development, with the greatest number and magnitude of differentially expressed genes between maternal care treatments, and enrichment for transcription factors regulating immune response in motherless early larvae. Metatranscriptomic data revealed fungi to be the most abundant group in the microbiome, with *Aspergillus* the most abundant in early larvae raised without mothers. Finally, integrative analysis between host transcriptome and metatranscriptome highlights several fungi correlating with developmental and immunity genes. Our results provide characterizations of the influence of maternal care on gene expression and the microbiome through development in a wild bee.

## Abstract (web summary)

A characterization of the metatranscriptome of the small carpenter bee across developmental stages shows the influence of maternal care on the microbiome and gene expression.

## Introduction

Parental care is an evolutionary adaptation in many species that has a profound impact on an individual’s development and species evolution^1^. Various forms and levels of parental care exist across mammals^2,3^, birds^4,5^, fish^6,7^, and insects^8,9^, directly enhancing the fitness of offspring by providing protection from predators and parasites, prolonged access to food supply, shelter, and social interactions^10,11^. Moreover, parental care has marked impacts on immunity and microbiome, such as the transfer of core microbes essential for offspring survival as observed in earwigs^12^, burying beetles^13^, African clawed frogs^14^, and mice^15^. The neurobiological and behavioral effects of parental care have been widely researched in social species, as it plays an essential role in the evolution of social behavior^16,17^. Abnormalities in behavior and brain chemistry have also been documented in other social mammals. Studies in rats have shown that low maternal care levels (characterized by low licking and grooming behavior) are linked to higher levels of stress reactivity in offspring^18^. In birds, where biparental care is prevalent, even the deprivation of one parent may result in permanent effects on the hypothalamic–pituitary–adrenal (HPA) axis, responsible for stress response and regulation of physiological processes^5^.

In particular, social insects are powerful model systems to explore the intricacies of parental care in the development of offspring. Bees demonstrate a highly diverse range of social behaviors, from obligately eusocial colonies with reproductive queens and foraging workers to solitary nests in which mothers do all tasks alone^19^. Such differences in social behavior can exert considerable influence on an organism’s microbiome and gene expression. Eusocial species typically receive their microbiome through horizontal transmission from nestmates, whereas solitary species acquire theirs horizontally from the wider environment^20^. Furthermore, facultatively social bee offspring reared with or without parental care display changes in gene expression patterns indicating that parental care can drive regulatory processes at the molecular level in offspring. For example, the adult small carpenter bee *Ceratina calcarata* showed more aggression and avoidance when reared without a mother, a behavioral change associated with changes in DNA methylation and gene expression patterns^21^.

Social and solitary insects also display differences in development. Obligately eusocial insects possess distinct castes, produced during development through differences in larval diet and developmental gene regulatory networks, ultimately resulting in different microbiome compositions for each caste^22,23^. While developmental castes are not present in facultatively social and solitary bees, recent studies have found that they exhibit clear changes in microbiome composition and richness across developmental stages^24,25^. Additionally, bees undergo complete metamorphosis and experience drastic changes in microbiome composition during defecation at the prepupal stage, which empties the gut, leading to an altered adult microbiome^25^. Gut microbiomes host a suite of taxa belonging to diverse groups such as bacteria, fungi, protists, viruses, and parasites (e.g., nematodes and arachnids) that individually and collectively impact host health and recently discovered important associations to host behavior via the gut–brain axis^26^. Microbiomes are, in turn, shaped by the environment and stressors associated with the host, which includes the influence of maternal care. For instance, breastfed infants develop healthy microbiomes supporting stronger immunological defenses and improved neurodevelopment^27^. In insects, earwigs reared without mothers had a reduced microbiome and suffered greater mortality than offspring obtaining essential anti-fungal bacteria from their mothers^12^. Hence, studies on the bee’s gut microbiome demonstrate an important role of microfauna in the healthy development of bee immunity^28^, digestion and nutrition^29^, behavior and cognitive ability^30^, reflecting the importance of host–microbiome species interactions^25,31^.

**Fig. 1:**
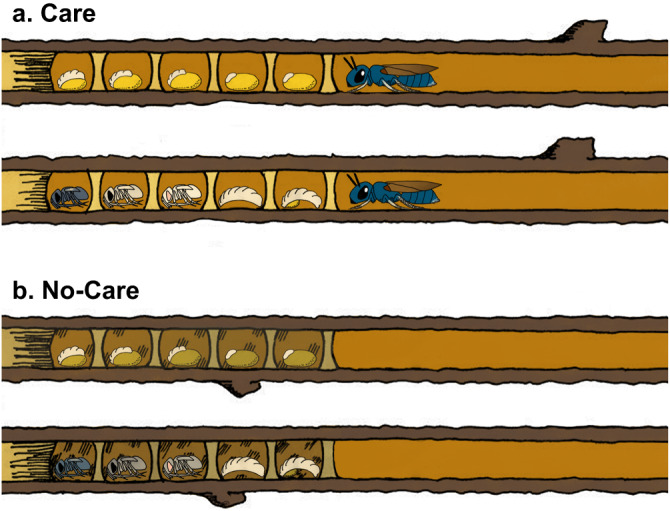
Development of *Ceratina calcarata* offspring in the presence or absence of mothers. **a** Mothers remain in nests throughout offspring development until adulthood, providing cleaning and nest guarding (care). **b** Absence of mothers results in dirtier nests due to overabundance of fungi (no-care). Illustration created in full by Katherine Odanaka.

Our model species, *C. calcarata* is a subsocial bee, which is characterized by prolonged maternal care, overlapping generations, and parent-offspring interactions^8,32,33^. The methylome, genome, and multiple transcriptomes of this species have been sequenced^34–36^. *C. calcarata* mothers are nest loyal, typically creating a single nest each year after emerging from overwintering in natal nests to mate in the spring^32^. Mothers excavate a linear tunnel in the pith of dead plant stems and sequentially lay one egg at a time on a freshly collected pollen ball before closing a cell by creating a wall from pith shavings^32^. As offspring develop, mothers continue to guard the nest and inspect the developing brood by breaking down and reconstructing cell walls and are, therefore subsocial^32^. This is done until offspring reach adult maturity, then they will continue to guard and feed adult offspring until late summer or fall^36^. Maternal care via guarding prevents fungal and bacterial outbreaks throughout offspring development (Fig. 1). While maternal care effects were explored in this species at the adult stage via gene expression patterns in the brain^21^, and its microbiome was explored through development^25^, we do not know what the changes in gene expression patterns are across this species’ development, nor do we know how maternal care impacts its developmental microbiome. This model species offers a natural experiment for studying the implications of maternal care for the connected phenomena of development and microbiome diversity.

Here we characterize the developmental transcriptome of *C. calcarata* and experimentally compare how the presence or absence of maternal care impacts gene expression patterns across development. Second, we examine these same offspring’s microbiomes across development and between maternal care groups. Finally, we integrate gene regulatory networks and co-expression with metatranscriptomic data across development and care groups to identify key candidate genes in offspring of various ages impacted by the absence of maternal and modified microbiomes. We predict dysregulation of offspring gene expression and altered metagenomic microbial communities in the absence of maternal care. This study provides unique insights into our understanding of offspring development, maternal care, stress, and immune response not only important for offspring health but also parental care in animals, including intergenerational inheritance more broadly.

## Results

### Developmental transcriptome

**Fig. 2:**
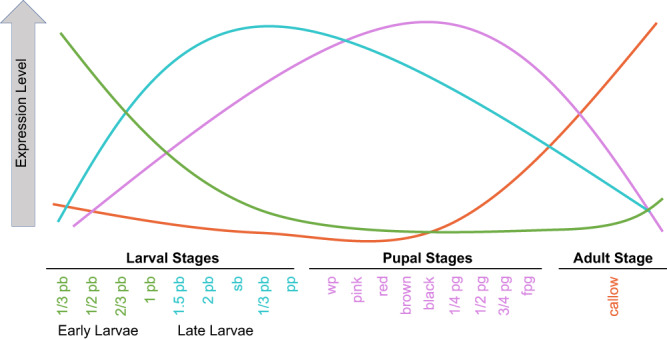
Generalized models of median gene expression underpinning the development of *Ceratina calcarata*. The four overall developmental stages include early larvae, late larvae, pupae, and callow (see Fig. S1 for reference).

**Fig. 3:**
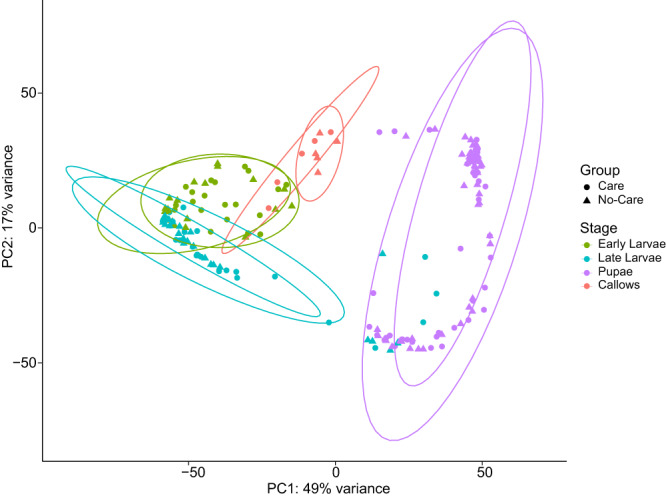
Principal component analysis of Ceratina calcarata developmental transcriptome in the presence or absence of maternal care. PCA plot of the four main developmental stages of *C. calcarata* in the presence of maternal care (care) and in the absence of maternal care (no-care). Ellipses for care and no-care groups are shown for each developmental stage.

Four generalized clusters of time course gene expression across *C. calcarata* offspring development were identified, summarizing the 19 individual stages into four overall developmental stages: (1) early larvae, (2) late larvae, (3) pupae, and (4) callows (Figs. 2 and S1; Supplementary Data 4). Overall, in 1957 unique differentially expressed genes (DEGs) were identified based on developmental stage. Early larval development had 340 DEGs uniquely upregulated when compared to other stages (Supplementary Data 5; Fig. S2). We also implemented multiple analyses (WGCNA^37,38^, DESeq2^39,40^, and MaSigPro^41^) to further identify recurrently well-supported DEGs (i.e., robust DEGs) for each stage as these DEGs likely play a critical role in development, which resulted in 156 robust DEGs in early larvae that include nucleolar GTP-binding protein 1 (*non1*), nucleolar protein 58 (*nop58*), and eukaryotic translation initiation factor 3 subunit C (*eIF3-S8*) (Supplementary Data 4). Enriched GO terms for all early larvae upregulated genes related to cell growth and processes, such as TORC2 signaling, regulation of the cell cycle, physiological development, and morphogenesis (Supplementary Data 5). Significant transcription factor (TF) enrichment determined by STREME^42^ found no significant motif enrichment in early larvae when compared to other developmental stages (Supplementary Data 27). Out of the 383 upregulated DEGs unique to late larvae, 37 robust DEGs encoded kinesin-like proteins, such as *KLP61F* and *KIF18A*, essential cytoskeletal motor proteins (Supplementary Data 5 and 11). DEGs upregulated in late larvae were enriched for several cell cycle regulatory pathways and digestive and metabolic processes (Supplementary Data 6). Further analysis using WGCNA network clustering revealed late larval DEGs formed three significant clusters enriched for physiological developmental processes and metabolic processes, but also stress response pathways (e.g., detoxification, cellular response to stress; Supplementary Data 12). Late larval DEGs were enriched for motifs accommodating TFs mainly belonging to C2H2 zinc finger and SMAD factors (Supplementary Data 27), three of which were also identified as DEGs and include mothers against dpp (*mad*), TF GAGA (*trl*), and pair-rule protein odd-paired (*opa*) (Fig. 3). GO enrichment of late larval motifs were mainly involved with cell differentiation and gene regulatory processes whereas TF DEGs were mainly involved with embryogenesis, development, and neurodevelopment (Fig. 3, Supplementary Data 28).

The pupal stage had 1010 uniquely upregulated DEGs, with 113 robust DEGs that include genes involved with nervous system development such as leucine-rich repeat-containing protein 24 (*LRRC24*), neural-cadherin (*CadN*), and Down syndrome cell adhesion molecule-like protein (*Dscam2*; Supplementary Data 5). GO enrichment for pupal development is related primarily to protein modification and neurodevelopmental processes such as central nervous system neuron development and neuron fate determination (Supplementary Data 6 and 12). Pupal DEGs were enriched for a single TF motif that binds a C2H2 zinc finger, *ZNF610* (Supplementary Data 27), which harbors diverse biological roles such as segmentation and oogenesis, and several molecular functions including protein binding and exonuclease activity (Supplementary Data 28). Callows had 224 uniquely upregulated DEGs and 78 robust DEGs, some of which encode mitochondrial enzymes, which include isocitrate dehydrogenase subunit betaC mitochondrial (*IDH3B*), NADH dehydrogenase iron-sulfur protein 42 C mitochondrial (*NDUFS4)*, and isocitrate dehydrogenase subunit gamma C mitochondrial (*IDH3G)* (Supplementary Data 5). GO enrichment all upregulated DEGs in callows related to eye development and structural organization (Supplementary Data 6), wing disc and head development, sensory perception, and several biosynthetic and metabolic processes (Supplementary Data 12). Unique callow DEGs were focused on RNA metabolism (e.g., tRNA metabolic processes) and protein transport (Supplementary Data 6).

### Effects of maternal care on the developmental transcriptome

**Fig. 4:**
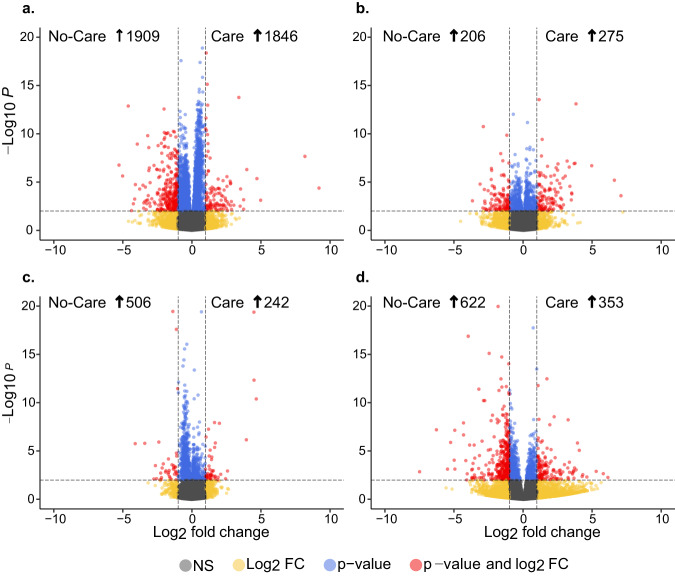
Volcano plot of the differentially expressed genes (red and blue) and not significantly differentially expressed (green and gray) at each developmental stage. **a** Early larvae, **b** late larvae, **c** pupae, and **d** callow stage of *C. calcarata* in the presence and absence of maternal care. The differentially expressed genes with log2 fold change greater than 1 are shown in red.

To experimentally assess the effects of maternal care on the developmental transcriptome, we performed transcriptional analysis on care and no-care developing brood. Overall, when care and no-care conditions were compared across all stages, 558 DEGs were unique to care, and 685 were unique to no-care (Supplementary Data 7). Cared-for bees were enriched for immunity, metabolism, and developmental processes, whereas no-care bees were mostly related to cellular development, cellular responses, and regulatory pathways (Supplementary Data 8). Comparisons between all care and no-care individuals revealed motif enrichment for a significant motif enriched only in cared individuals that binds the TF button-head (*btd*), which is involved with head segmentation (Supplementary Data 27). Clustering of two principal components (PC1 49% and PC2 17%) explained 66% of the variance in expression across the developmental stages in the presence and absence of maternal care (Fig. 3). No-care treatments resulted in differential gene expression (>4000 DEGs) between care and no-care conditions at each developmental stage (Figs. 4 and S3; Supplementary Data 9). In total, 4953 unique DEGs (Supplementary Data 9) were identified across all four stages and maternal care groups, of which 898 were uniquely identified as hub genes in our WGCNA network analysis (Supplementary Data 13).

Early larvae had 3755 upregulated DEGs due to differences in maternal care, with more DEGs identified in no-care early larvae (Fig. 4a). Negative binomial and network analyses identified venom allergen 3 (involved in the immune response) and digestion inhibitory genes such as chymotrypsin inhibitor and lysosomal aspartic protease to be upregulated in no-care early larvae (Supplementary Data 9 and 13). Inhibition of peptidases such as chymotrypsin has been shown to interfere with insect metabolism^43^, whereas proteases are involved with insect immunity against pathogenic invasions^44^. Conversely, defensin 1 was upregulated in care early larvae, an important gene for antimicrobial peptides in insects^45^ (Supplementary Data 9). Gene Ontology (GO) revealed genes upregulated in the care early larvae were enriched for immune processes (e.g., natural killer cell activation, phagocytosis, and T cell activation). No-care early larvae were instead enriched for protein movement and reproductive purposes (e.g., meiotic cell cycle, embryo development, and protein targeting; Supplementary Data 10). GO enrichment for the top three significant modules from WGCNA results for each maternal care group, and stage showed that maternal care presence leads to regulatory pathways focused on cellular development and gene regulation, but also immune defenses and metabolism (e.g., response to bacterium and monosaccharide metabolic process; Supplementary Data 14). WGCNA modules in no-care early larvae were conversely enriched for antigen processing, epithelial cell proliferation, and several catabolic processes (Supplementary Data 14). Furthermore, among all maternal care+stage group comparisons, significant TFs were only detected in no-care early larvae DEGs which were enriched for a motif that binds to the chromatin-linked adapter for MSL proteins (*CLAMP*) and protein hairy (*h*) (Table 1; Supplementary Data 27). GO enrichment of this motif is related to several developmental processes, such as brain development and chromatin assembly (Supplementary Data 28).

**Table 1:** Enriched transcription factor binding motifs.

			Category						
TF gene name	Role	Ref.	Care	No-care	Early larvae	Late larvae	Pupae	Callow	
btd	Head segment development	^110^	0.013						
ZNF148	Muscle differentiation	^111^				0.046			
ZNF610	DNA methylation, carcinogenesis	^112^				0.046	0.0045		
Mad^a^	Eye/wing development	^113,114^				0.031			DEG
Clamp	Chromatin accessibility, male dosage compensation	^115^		0.019	0.019	0.021			
Trl	Embryogenesis, oogenesis, eye development	^116^				0.0046			DEG
Opa	Embryogenesis, circadian rhythm, adult head development, neural development, behavior	^117^				0.0052			DEG
h	Embryo patterning, heart development, hypoxia tolerance	^118,119^		0.047	0.047				DEG

Transcription factor binding motifs significantly enriched in upstream regions of upregulated DEGs across maternal care and stage groups (see Supplementary Data 27 for category comparisons). Transcription factor names are presented with a brief description of regulatory roles. *Q*-values which is the minimal false discovery rate for multiple-testing correction, are shown in cells; empty cells indicate no significant (*E* > 0.05) motif enrichment identified in the category. Associated transcription factors identified as a DEG in this study are labeled as DEG.

^a^If multiple motifs are matched to the same transcription factor in the same category, a higher *q*-value is presented.

No-care late larvae had 206 upregulated DEGs (Fig. 4b), including genes involved with homeostatic compensation mechanisms such as potassium voltage-gated channel protein (*shab*). DEGs upregulated in late larvae were enriched for reproductive processes and physiological development, including mesoderm development and rhythmic process (Supplementary Data 10). DEGs upregulated in no-care late larvae were enriched for ion transmembrane transport, protein import, and regulation of transporter activity (Supplementary Data 10). Care and no-care late larvae shared biological processes enriched for light detection, catabolic processes, and stress response (Supplementary Data 14).

Pupae reared without mothers had 506 upregulated DEGs, including LRRC15, involved in insect immune response^46^ (Fig. 4c). GO enrichment of upregulated DEGs included protein phosphorylation in care pupae or protein dephosphorylation in no-care pupae (Supplementary Data 10), whereas network clustering found identical modules in care and no-care pupae enriched for compound eye development, digestive system development, and regulation of mitotic cell cycle (Supplementary Data 14).

**Fig. 5:**
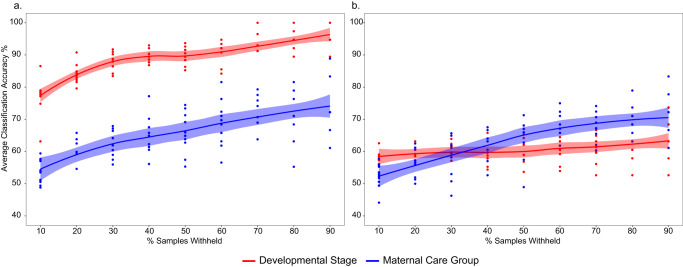
Overall average performance accuracy for each model is shown for separate random forest classifiers (RFC) that test if the overall developmental stage or maternal care group can predict samples. RFCs assign samples either to the overall developmental stage or maternal care group using either **a** gene expression data or **b** metatranscriptomic abundance data. RFC training was done from 10% to 90% of samples withheld. Models using gene expression data, on average, performed better in classifying samples to developmental stages or care groups compared to models using metatranscriptomic data (all domains included). Error bars were generated using the loess smoothing curve method. Full results for gene expression data are shown in Supplementary Data 18 and 19 for the maternal care group and overall developmental stage, respectively. Full results for metatranscriptomic data are shown in Supplementary Data 20 and 21 for the maternal care group and developmental stage, respectively.

Among callow offspring, 622 DEGs were upregulated in no-care, including hexamerin, which is known to be involved in immune response^47^, and calcium/calmodulin-dependent protein kinase II (CAMKII), known to be involved with learning and memory^48^ (Fig. 4d). GO enrichment for care callows include protein modification processes and several physiological developmental processes such as mesoderm development/formation, exocrine system development, salivary gland development, and gland morphogenesis (Supplementary Data 10). No-care callows GO enrichment include terms such as RNA modification, tRNA modification, response to hormone, and secretion (Supplementary Data 10). Furthermore, GO enrichment of WGCNA network clustering for care and no-care callow DEGs revealed major physiological development, such as wing disc development, and several metabolic and biosynthetic processes (Supplementary Data 14). However, WGCNA analysis also revealed that care callows were enriched for unique physiological processes such as locomotion and metamorphosis, whereas no-care callows were uniquely enriched for cell response pathways such as cAMP-mediated signaling and sensory perception of pain (Supplementary Data 14). Interestingly, no significant motif enrichment was identified in callows in any of the developmental stage comparisons or stage + care group comparisons. Finally, RFC models using transcriptomic data revealed that gene expression data was better at predicting offspring based on their developmental stage (average accuracy of 94%; Supplementary Data 19), rather than the maternal care group (average accuracy of 71.6%; Fig. 5a; Supplementary Data 18).

### Effects of development and maternal care on the microbiome

**Fig. 6:**
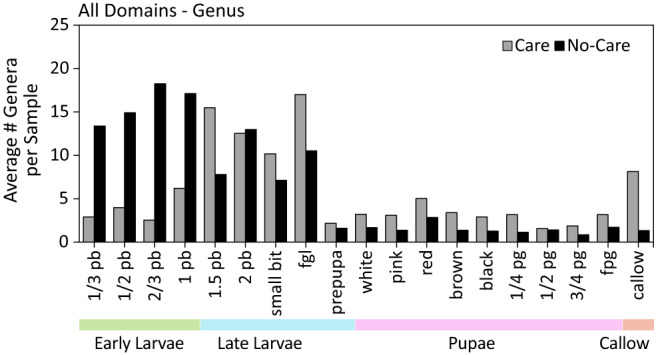
The average number of microbiome genera per sample across all domains for each individual developmental stage and maternal care group (*N* = 190). See Supplementary Data 2 individual for developmental stage names.

Metatranscriptomic analyses using metaSPADES^49^ identified 842 microbial genera across developmental stages and care groups. The most dominant microbial group is fungi, contributing to approximately 85% of the microbiome composition, followed by bacteria (8%) based on contig abundance (Supplementary Data 15). Absence of mothers resulted in a significant difference in taxa composition (Bray–Curtis PERMANOVA *F* = 7.72, d*f* = 3, *p* = 0.001, Fig. 6) and alpha diversity across developmental stages (Shannon ANOVA *F* = 1.26, d*f* = 3, *p* = 8.52e−09; Fig. 6; Supplementary Data 16). Metatranscriptomic data performed better in predicting the maternal care group (average accuracy of 70%; Supplementary Data 20) rather than the developmental stage (average accuracy of 61%; Fig. 5b; Supplementary Data 21), even when using bacterial, fungal, or bacterial+fungal genera only (Fig. S4; Supplementary Data 20 and 21). Furthermore, there is a striking difference in microbiome composition from fully grown larvae and prepupae stages across all individuals (Fig. 6) and within groups (Fig. S5).

Maternal presence significantly affected alpha diversity (Shannon ANOVA *F* = 16.75, d*f* = 1, *p* < 0.01) and community composition (Bray–Cutis PERMANOVA *F* = 10.43, d*f* = 1, *p* = 0.001) (Supplementary Data 17) during early larvae development, with greater diversity in no-care offspring. *Aspergillus* was the top fungal genus that dominated the community composition in early larvae in both care and no-care groups (Supplementary Data 22). However, *Aspergillus’* relative abundance was significantly greater in no-care individuals (28%) relative to offspring reared with mothers (16%; two-tailed *t*-test *t* = −4.58, d*f* = 38, *p* < 0.01). *Aspergillus* was also identified in SIMPER^50^ analysis as a top 10 contributor to metagenomic composition dissimilarity (Supplementary Data 23) and was the only fungus identified as a top 10 feature during random forest classification (RFC) model testing for maternal care influence when using all taxon groups using the *randomForest* package in R^51^ (Supplementary Data 20). Other top genera based on relative abundance and identified by SIMPER were also fungi, including *Ascosphaera* and *Paecilomyces*, which also had greater abundances in no-care early larvae (Supplementary Data 22 and 23). Negative binomial distribution analysis (NBDA) obtained from *DESeq2* analysis corroborated our earlier findings, with significant overrepresentation of fungi in early larvae (Supplementary Data 24), as well as a significant WGCNA cluster of hub fungi in no-care early larvae (Supplementary Data 26). In late larvae, taxa composition and richness did not show significant differences between care groups (Supplementary Data 17). Indeed, the top genus, *Aspergillus*, was not significantly different in abundance between no-care and care individuals (two-tailed *t*-test *t* = 1.27, d*f* = 48, *p* = 0.212).

**Fig. 7:**
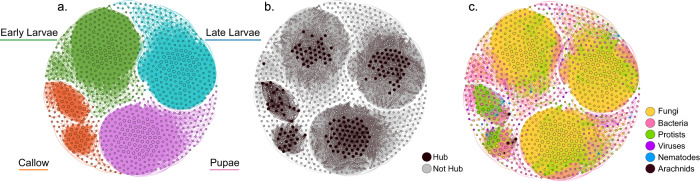
Significant WGCNA modules identified at the genus level. **a** Shows each developmental stage (two modules for early larvae, one for late larvae, one for pupal, and two for callow). In **b**, hub genera are highlighted in dark gray, and **c** the module compositions are colored by domain. Nodes are sized according to whether they are a hub genus (but also colored in diagram B).

Pupal taxa richness was significantly different from the larval stages but were not different from callows (Supplementary Data 16). Interestingly, while pupae defecate and lose most of their gut microbiota, there was a significant difference in taxa richness and composition between care groups (Supplementary Data 17), with different top genera depending on maternal care presence (Supplementary Data 22). *Aspergillus* and *Ascosphaera* were significantly overrepresented in care pupae and suggest maternal presence may be reintroducing fungal taxa during the pupal stage (Supplementary Data 24). WGCNA network analysis found the top fungal cluster from the larval stages also evident in pupae (Fig. 7, Supplementary Data 25).

The top genera in callows were more diverse in taxa richness, and composition differed significantly between care groups (ANOVA, *F* = 11.69, d*f* = 1, *p* < 0.001; Bray–Curtis PERMANOVA *F* = 2.36, d*f* = 1, *p* = 0.015; Supplementary Data 17). For instance, callows in the presence of mothers were dominated by a mix of fungi (e.g., *Aspergillus* and *Ascosphaera*), viruses (e.g., *Nepovirus* and *Betacoronavirus*), and protists (e.g., *Gregarina* and *Plasmodium*; Supplementary Data 22). Conversely, no-care callows were mostly dominated by bacteria, including notable genera such as *Pseudomonas*, *Acinetobacter*, and *Burkholderia*. There was no significant overrepresentation of taxa between callow care groups or between callows and other developmental stages (Supplementary Data 24). Interestingly, WGCNA network analysis found unique but smaller modules for callows, which included clusters of primarily bacteria and protists (Fig. 7; Supplementary Data 26). As such, callows differed the most in module clustering and composition (Fig. 7). The presence of mothers contributes to slightly greater taxa abundances, as suggested by hub genera identified in cared callows but not in uncared callows (Supplementary Data 25).

### Comparison and integration of gene expression and metatranscriptomic data

**Fig. 8:**
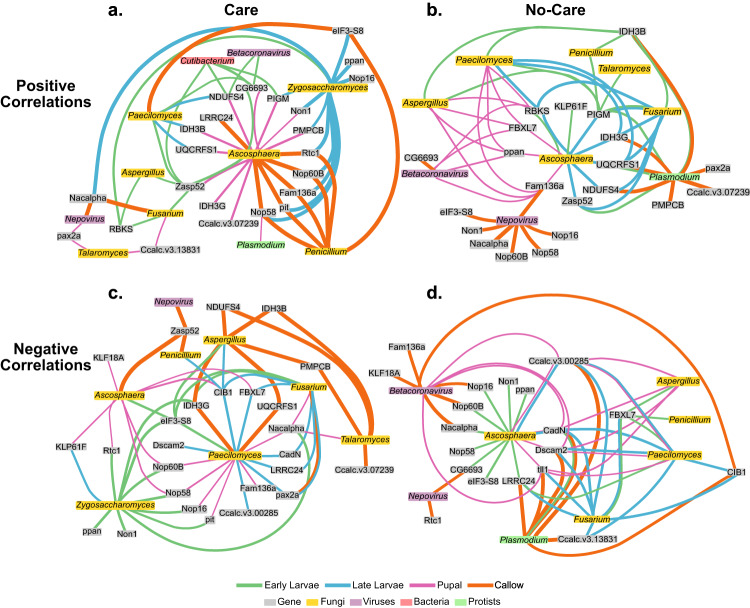
Top positive and negative correlations between top genes and top genera. Correlations shown are at least the top 15 positive or negative correlations throughout the developmental stages and are separated into **a** positive correlations for care, **b** positive correlations for no-care, **c** negative correlations for care, and **d** negative correlations for no-care offspring. Connections between nodes are colored to indicate the developmental stage, and the thickness of the line indicates the strength of the correlation.

We used the *mixOmics*^52,53^ R package to run an integrative correlation analysis of gene expression and metatranscriptomic abundance data which revealed that *Ascosphaera* held the majority of top positive correlations in cared-for offspring, primarily in pupal and callow developmental stages (Fig. 8a). *Ascosphaera* correlated with mitochondrial respiratory genes and enzymes (i.e., *UQCRFS1*, *IDH3B, IDH3G*) in cared pupae, and correlated with ribosomal developmental genes (i.e., *non1*, *nop16*, and *nop58*) in callows. Similarly, top positive correlations in no-care individuals also identified *Ascosphaera* as a central fungus correlating with several developmental genes, but primarily across the larval and pupal stages (Fig. 8b). Surprisingly, instead of several strong correlations between *Aspergillus* and developmental genes in no-care early larvae, *Aspergillus* only correlated with a few genes involved with mitotic development (i.e., *FBXL7*, *ppan*, and *fam136a*) in pupae, and the mitochondrial enzyme *IDH3B* and the glycotransferase *PIGM* in early larvae. Callows were the most different, with genes correlating with the viruses *Betacoronavirus* and *Nepovirus*, and the protist *Plasmodium*, reiterating previous results that a change in microbiome composition is most notable at the callow stage.

Among the top negative correlations in cared individuals, *Paecilomyces* was the top fungus with the most negative correlations across all four developmental stages (Fig. 8c). Similar to *Paecilomyces, Fusarium* also negatively correlated with several genes across the four stages, whereas the remaining fungi primarily correlated with only one or two developmental stages. In contrast, *Ascosphaera* held the most negative correlations in no-care offspring, with correlations to ribosomal and mitochondrial genes mainly in early larvae (Fig. 8d). All fungi negatively correlated with genes from no-care early to pupal individuals, whereas protists and viruses negatively correlated with developmental genes primarily in no-care callows.

## Discussion

This study characterized the regulation of gene expression and the metatranscriptome of *C. calcarata* across development and in the presence or absence of maternal care. The developmental transcriptome reveals gene expression according to development and maternal care, with a stronger baseline gene expression profile attributed to development. We found that early and late larval stages exhibit a diverse microbiome with fungi predominating. In the absence of maternal care, early larvae are most susceptible to fungal and bacterial overabundance, whereas later developmental stages have a much-reduced microbiome. Finally, we found interesting correlations between fungal taxa and candidate bee developmental genes that are potentially involved with immunity. To our knowledge, our study is the first to provide metatranscriptomic insights into the relative role of maternal care on offspring development and a foundational framework for the developmental microbiome, a critical component of bee health.

### Time course developmental gene expression of *C. calcarata*

Genes encoding ribosomal protein subunits, nucleolar proteins, and translation initiation TFs were found to be upregulated during the early larval stage of *C. calcarata*. Ribosome genesis is central to larval development in insects and has an important role in early developing endoderm of zebrafish^54,55^. Ribosomal proteins are essential during periods of cell proliferation and development^56^. Additionally, kinesin-like protein-encoding genes central to normal mitotic processes (*KLP61F*) are upregulated in late larvae. The disruption of such genes has been shown to result in larval lethality^57^.

Neurodevelopmental genes are upregulated in the pupal stage, such as *NLGN1*, *LRRC24*, and *cadN*, which are central to synapse function and cognition^58–60^. Additionally, the upregulated genes include an essential TF (*sox15)*, which is mainly expressed in external sensory organs’ socket cells and has a role in the proper electrophysiological function of *Drosophila melanogaster’s* mechanosensory organs^61^. Tramtrack (*Ttk*), which is an important transcriptional repressor for pupal *Drosophila* neurodevelopment and has a role in caste differentiation of ants, is upregulated in pupal *C. calcarata*^62,63^. Overall, pupal gene expression found across *C. calcarata* is mainly involved in neurodevelopment and mechanosensation.

During the callow stage, there are several upregulated genes involved in oxidative phosphorylation, including *cox6a1* (cytochrome c oxidase subunit 6A1) and *NDUFA7* (NADH mitochondrial complex I dehydrogenase 1 alpha subcomplex subunit 7). Flight muscles of *D. melanogaster* are rich in mitochondria^64^; the upregulation of mitochondrial enzyme-encoding genes indicates the essential role of wing development in newly emerged *C. calcarata* callow. Overall, upregulated callow developmental genes are mainly involved in wing muscle development.

Across all developmental stages, TF enrichment was most often associated with the late larval stage and consisted mainly of C2H2 zinc finger TFs. C2H2 zinc finger TFs are involved with growth and play a role in stress response^65^, which may be indicative of rapid development that occurs prior to pupation^32^ and a sensitivity to external stimuli as demonstrated by GO enrichment of late larval motifs relating to signal transduction, cell communication, and several physiological developmental processes.

Pupae were enriched for *ZNF610*, which has a putative role in DNA methylation^66,67^. Insect pupae are sensitive to external stimuli (e.g., temperature), which can epigenetically alter gene expression resulting in polyphenism. While DNA methylation has been linked to caste differentiation in social bees (i.e., honeybee queens and workers), the lack of caste differentiation in solitary bees suggests that DNA methylation plays a more prominent role in the regulation of gene expression and transcription^68^. Finally, the lack of TF enrichment in callows compared to other developmental stages or between callow maternal care groups advocates for similar, basic biological processes that occur at the adult stage post-development.

### Maternal care has the strongest effect on gene expression during early larvae development

Our results indicate that maternal care deprivation affects gene expression across the development of *C. calcarata*, most significantly during early larval development. In this stage, with the absence of maternal care, upregulated genes encode trehalase, venom acid phosphatase (*acph-1*), and venom allergen 3. Trehalase (which converts trehalose to glucose) is upregulated in the absence of maternal care, as found in response to increased oxidative stress in mice^69^. Additionally, venom acid phosphatase is a major allergen in *Apis mellifera* and is a component of bee venom, which has a protective property in bees^70,71^. These results suggest that the absence of mothers during early development is associated with genes involved in mediating immune and oxidative stress responses. Parental care has been shown to play an important role in egg survivorship and early larval development in other insects with facultative parental care. For instance, European earwig mothers will increase their care load depending on pathogen exposure within their nests, accordingly, such as returning to nests more often for egg care^72^. In burying beetles (*Nicrophorus vespilloidesi*), larval survivorship and growth rates were significantly higher in the presence of parental care^13^.

On the other hand, among downregulated genes is eukaryotic translation initiation factor 3 subunit E (*eIF3-S6*) and various ribosomal protein subunits. These results indicate that maternal care deprivation could be linked to downregulating genes essential for translation initiation and potentially affect cell proliferation and development. Additionally, no-care early larvae were the only group+stage with DEGs enriched for a significant motif that binds to a C2H2 zinc finger and a zipper-type TF, with enriched roles in TF activity, brain, and eye development. While we might expect stressed offspring to show term enrichment directed to immunity, regulation of transcription activity is itself a response to stress and serves as a defensive mechanism by regulating several genes in order to maintain cellular integrity, prioritize development, or changes in cellular behavior^73^.

Several other genes demonstrated changes in expression due to maternal care, but a notable change in expression occurred in a potassium voltage-gated channel protein (*shab*) which was upregulated in no-care late larval samples. It has been shown to be upregulated as a compensatory mechanism in response to loss of Ca^2+^-dependent K^+^ channel conductance in *D. melanogaster*^74^. It could be that *shab* upregulation is part of a homeostatic mechanism because of maternal care deprivation. Pupae reared without mothers had upregulated genes, such as *LRRC15*, whose transcription has been shown to be enhanced in response to neonicotinoids in honeybees^75^. There were also many more significantly upregulated DEGs in no-care callows, which include hexamerin, a gene found to be upregulated in the leafhopper *Circulifers haematoceps* as part of an immune response and bacterial infection controlling mechanism^47^. Additionally, CAMKII is upregulated in no-care conditions; upregulation of this gene is known to result in memory impairment in *Drosophila*^48^.

RFC testing demonstrated that gene expression was highly accurate in predicting the developmental stage of offspring when compared to predicting the maternal care group, suggesting that while mothers do play a role in gene regulation during early larval development, the absence of mothers does not arrest the full development of *C. calcarata* offspring.

### Fungi pose a risk of developing early larvae without maternal care

We expected a change in metatranscriptomic composition across development and between maternal care groups, and we observed a significant difference in taxa richness, specifically between early and late larvae versus pupal stages. Interestingly, we found that while transcriptomic data groups prepupae with late larvae based on gene expression, microbiome composition shows a greater similarity between prepupae and the pupal stages. This difference is likely due to prepupal gene expression patterns most similar to late larval stages as it is a pre-metamorphic state^25^, however, defecation begins in prepupae^32^, and emptying of the gut would result in a similar, decreased microbiome composition in prepupae and in pupae.

Our RFC models showed that the metagenome is slightly better at predicting the maternal care group rather than the developmental stage for offspring, highlighting the importance of nest cleaning and rebuilding of nest cell walls by mothers^32^ to keep fungi and bacteria at a minimum. Studies have shown that *Ceratina* nests without mothers had a greater percentage of infested cells and greater brood mortality^76^.

The fungi *Aspergillus* consistently showed up as a highly abundant and persistent genus in *C. calcarata* in the early larval stages. *Aspergillus* was dominant in early larvae samples reared without mothers, along with other plant-associated and potentially pathogenic fungi such as *Paecilomyces* and *Ascosphaera. Aspergillus* is associated with environmental diseases such as plant molding and food rot^77^. The most well-known disease by *Aspergillus* in bees is stonebrood disease, in which *Aspergillus flavus* infects honeybee larvae causing mortality and mummification^78^. *Ascosphaera*, a fungus that causes chalkbrood disease in honey bees^79^, was also found overrepresented in no-care early larvae. Although direct effects of *Aspergillus* or other fungal pathogens on *C. calcarata* larval mortality remain to be tested, *Aspergillus* is known to cause pollen spoilage and increased mortality in solitary ground-nesting *Nomia* alkali bees^80^ and *Diadasia* chimney bees^81^, and the strain *Ascosphaera proliperda* caused chalkbrood disease in *Megachile* leaf-cutting bees and *Osmia* mason bees^82^. It is evident that *Ceratina* mothers actively keep their brood cells clean to avoid the spread of fungal pathogens and pollen spoilage^32,76^ and that this active cleaning appears to be most important in the early developmental stages (Fig. 6).

### Key correlating genes with fungi

Integrative analyses revealed that *Ascosphaera* and *Paecilomyces* consistently harbored the most associations with host offspring gene expression across the four developmental stages and between care groups. *Ascosphaera* was the central fungus with the most positive correlations in both care and no-care treatments, but interestingly there were many more correlations between *Ascosphaera* and developmental genes when mothers were present. This suggests that even in the presence of mothers, offspring are reacting to a fungal and highlights the need for future studies to explore the pathogenic effects of *Ascosphaera* on offspring survivorship in *C. calcarata*. In callows, *Ascosphaera* correlated with pitchoune (*pit*), potentially involved with ribosomal biogenesis and protein synthesis^83^, and in pupae with isocitrate dehydrogenase *IDH3B*, involved with amino acid metabolism^84^; these genes were not positively correlated with *Ascosphaera* under no-care conditions. Although the effects of *Ascosphaera* are unclear in solitary wild bees, it is known to infect the later larval and pupal stages of honey bees by germinating in their digestive tract and mummifying larvae and pupae^85^. We speculate that *Ascosphaera* induces a stress response in *C. calcarata* in cared and no-cared larvae and pupae, leading to increased energy production and mitotic and ribosomal development to offset developmental stress. Conversely, *Ascosphaera* positively correlated with the cellular respiratory gene *UQCRFS1* in pupal stages in cared treatments, but a lack of mothers resulted in the same correlation during the late larval stages. The *UQCRFS1* gene is involved with energy production, a process that is often increased under stress conditions^86^, and this corroborates the results found in honey bee larvae infected with *Ascosphaera apis*, where energy metabolism was elevated^87^. Furthermore, in no-care offspring, *Ascosphaera* negatively correlated with several ribosomal developmental genes in the early larval stage. This finding suggests that in a stressed environment, ribosomal biogenesis—which requires abundant energy metabolism - is diminished. This might be due to increased energy expenditure in other processes in no-care early larvae, such as the positive correlation between *Ascosphaera* and *RBKS*, a gene involved with carbohydrate metabolism. Indeed, bees feed on a carbohydrate rich diet, and *C. calcarata* early larvae are known to feed constantly on their pollen mass provision allowing them to develop into pupae in little under a month^32^. Future functional work on solitary bee offspring is needed to better understand how offspring respond genetically and physiologically when faced with *Ascosphaera* infections, with or without maternal presence, as our results suggest that certain fungi may be adept at surviving in nests despite cleaning and guarding by mothers.

*Paecilomyces* is a fungus found in soil and decaying plant material and produces secondary metabolites that can be toxic to insects^88^. As such, the presence of *Paecilomyces* still induces a stress response even in brood reared with mothers. For instance, while *Paecilomyces* negatively correlated with *Dscam2*—which has a role in neuronal development^89^ and immunity^90^—in no-care late larvae, it also negatively correlated with *Dscam2* in cared-for late larvae, suggesting that irrespective of maternal care, regulation of immunity genes in offspring are faced with a cost due to fungal stressors. Additionally, *Paecilomyces* negatively correlated with other neurodevelopmental genes in care and no-care late larvae suggesting that neurodevelopment is deficient in the presence of *Paecilomyces* and may lead to nervous system dysfunction, as was found to occur in ghost moth larvae infected with *P. hepiali*^91^. Future functional studies on fungal infections during development will be necessary to fully understand how these fungi impact non-apid bee survival and how maternal presence may alleviate the stress of fungal stressors.

Whereas previous studies focused only on the effects of maternal care in adult *Ceratina* gene expression^21^ or only on the *Ceratina* developmental microbiome without maternal absence^25^, our study uniquely analyzes the changes in gene expression patterns and microbiome composition across developmental stages in the presence and absence of maternal care. Arsenault et al.^21^ found that the removal of mothers during the larval stage resulted in more aggression and avoidance in adults as a result of changes in gene expression patterns, with notable changes in genes related to neuronal function and metabolism. Although we did not explore behavior in our study, we found that maternal care had the greatest impact during early larvae development with the greatest number and magnitude of DEGs, also involved primarily with neurodevelopment, but also involved ribosome biogenesis. Microbiome analyses corroborated the finding that maternal care plays an important role during early larvae development, as fungal pathogens such as *Aspergillus*, had the greatest abundance in the absence of maternal care, but was much reduced in offspring with mothers present. This differs from Nguyen et al.^25^, which did not find *Aspergillus* as a dominant fungus in their *Ceratina* samples but did find other fungi to be prominent, which were also found in high abundance in our study, such as *Ascosphaera* and *Penicillium*. Machine learning analyses of the full metatranscriptomic dataset found that gene expression was better able to predict offspring developmental stage than maternal care group, whereas the opposite was true for microbial communities, reinforcing the utility of hologenomic datasets to examine the relative roles of genes and environment. Potential interdependence between host gene expression and microbiome datasets highlights important interactions, such as the fungi *Ascosphaera* and *Paecilomyces*, as prominent stressors and the role of maternal care in maintaining development. This study provides foundation developmental and microbiome characterizations of a wild bee and network syntheses to develop hologenomic insights into early childhood development and health.

## Methods

### Brood rearing and sample collection

*C. calcarata* nests were collected from sumac and raspberry stems in Toronto, Canada (43.7735° N, 79.5019° W). Care and no-care treatments were raised in the respective presence and absence of maternal care to each of the 19 individual developmental stages based on Rehan and Richards^32^ (Supplementary Data 2). Following the protocol design in Arsenault et al.^21^, care and no-care offspring were reared in the lab at 23 °C and 50% relative humidity to each of the focal developmental stages before assay with and without the presence of mothers, respectively. All samples were flash-frozen in liquid nitrogen and stored in a −80 °C freezer.

### RNA extractions, library construction, and sequencing

RNA was extracted using Qiagen RNeasy mini kit from whole body samples of five individuals from each stage in both care and no-care treatments. In total, 190 samples of larvae, pupae, and adults (from 140 nests) were sequenced in this study. For this experiment, there were 95 care group samples and 95 no-care group samples used. The pupae and adult samples used were all females. RNA samples were then sent to Genome Quebec for library preparation using the TruSeq DNA PCR-free method and paired-end sequencing using the Illumina NovaSeq 6000 platform. All samples were sequenced to an average depth of approximately 33× coverage to 100 bp (Supplementary Data 3). The reads were then aligned to the *C. calcarata* reference genome (PRJNA791561) using STAR^92^. Adapter contamination was less than 0.2% in reads and STAR accounts for poor quality ends of reads during alignment.

### Differential gene expression analysis

Normalized counts and DEGs across developmental stages and between care and no-care treatments were obtained using *DESeq2*^39,40^. Heatmaps were generated using *pheatmap*^92,93^ in R based on variance-stabilizing transformed data from the *DESeq2* package. PCA plots were generated based on variance-stabilized transformed data. To explore changes in gene expression for DEGs between care and no-care groups, volcano plots were generated using *EnhancedVolcano* package^94^. As recommended in *maSigPro* user’s guide^41^, *EdgeR* was used to obtain normalized library counts without any CPM filtering in order to keep all genes for analysis^95^. To survey gene expression over the developmental stages, these data were then inputted into *maSigPro* to obtain generalized linear models in which the adjusted *p*-value is less than 0.05. Additionally, *maSigPro* uses a linear step-up Benjamini–Hochberg false discovery rate procedure, in which the adjusted *p*-value is less than 0.05^41,96^. A higher regression model was used (degree = 18) to account for the 19 individual developmental stages over time, with a backward stepwise regression to identify gene profile differences. Gene clusters obtained from *maSigPro* were used to generate generalized linear plots, which allowed the 19 individual developmental stages to be merged into four overall developmental stages (early larvae, late larvae, pupae, and callows) that will be used for further analysis (Supplementary Data 2, Figs. 2 and S1). The GO enrichment of the gene lists obtained using *DESeq2* and *maSigPro* were then functionally annotated using *TopGO* with enriched terms identified by Fisher’s exact tests with a p-value less than 0.05^97^.

### Gene overrepresentation and cluster analysis

To identify key co-occurring DEGs, normalized gene expression data for the complete dataset (characterized by stage and maternal care group) from *DESeq2* was used for weighted gene co-expression analysis (WGCNA) using the *wgcna* R package^37,38^. The samples were clustered using the hclust distance method and using a soft thresholding power of 6, module size of 50, and unsigned network type, WGCNA analysis was conducted, and no outlier samples were detected^37,38^ (Fig. S6A). In this study, we have focused on the top three significant modules (based on the highest positive correlation values and *p* < 0.05) for each stage and group. GO enrichment for these genes was obtained as well. Genes were classified as a hub gene in the module if the absolute gene significance value (GS) was≥0.2 and absolute module membership values (MM) were ≥0.9 according to cut-off values implemented in previous studies utilizing WGCNA^98,99^; the MM cut-off was increased to 0.9 from 0.8.

### Taxonomic classification

Unmapped transcriptomic reads were used to characterize the holobiome of each offspring and for the taxonomic classification of microbial communities. First, metaSPADES (version 3.10.1), a package from the SPAdes toolkit^49^ was used to obtain contigs from the reads. Contig assembly was done for paired-end reads using default kmer settings (-k 21, 33, 55, 77). Contig files for 190 samples were produced, then BLASTed using blastn^100^ (version 2.12.0) against the non-redundant nucleotide *nt* database, setting parameters -max_target_seqs and -max_hsps to 1 in order to extract only the top alignment hit for each contig query. We removed contigs shorter than 200 bp and contigs mapping to environmental bacterial contaminating genera *Sodalis* or *Wolbachia*^101^ (**3**). Next, NCBI Entrez Direct E-Utilities *efetch* (version 15.3) (https://www.ncbi.nlm.nih.gov/books/NBK25499/) was used to extract full lineage information for the remaining contigs (e.g., kingdom, phylum, class, order, family, genus, species) based on the BLAST hit taxonomy ids (i.e., ‘taxids’). Contigs were kept if they matched any one of the six groups of interest at the genus level: arachnida, bacteria, fungi, nematoda, protista, and viruses. Then, we further filtered the contigs using a minimum relative abundance threshold of 0.1% within a sample for each group of interest. Genera were considered top taxa for each category (i.e., developmental stage + care group) if they were above 1% relative contig abundance for that category.

### Diversity analyses

We tested for significant differences in genus dissimilarity and richness using Bray–Curtis dissimilarity and Shannon-Wiener diversity indices, respectively. First, we used the R package *vegan*^102^ to test Bray–Curtis dissimilarity on the metatranscriptomic dataset, transformed using the total method in DECOSTAND, to infer any significant differences across the four developmental stages (early larvae, late larvae, pupal, callow). PERMANOVA via the ADONIS2 method from *vegans* was used to determine if the resulting dissimilarity was significant between stages. To ensure PERMANOVA was not violated due to unequal variation among groups, the BETADISPER method was used to test for the significance of the PERMANOVA result; it is violated if an ANOVA test on the BETADISPER result is significant. If valid, Tukey’s Honest Significant Difference (HSD) via the TUKEYHSD method was implemented to identify groups that were significantly different from each other. Next, we ran Shannon-Wiener to test for significant differences in alpha diversity across all developmental stages. ANOVA via the AOV method was used to determine the significance of alpha diversity, followed by Tukey’s HSD to identify significantly different groups. We also ran the Kruskal–Wallis test on Shannon–Wiener alpha indices as a second non-parametric test to identify significantly different groups. First, Bartlett’s test of variance using the BARTLETT.TEST method was implemented to ensure variance across samples was equal. If not significant, the Kruskal–Wallis using the KRUSKAL.TEST method was applied. If significant, Dunn’s test using DUNNTEST from the R package *FSA*^103^ was used to identify significantly different groups. Next, we ran the above analyses to test for significant differences in dissimilarity and alpha diversity for each stage between maternal care groups (e.g., care: early larvae vs. no-care: early larvae). Finally, we ran a similarity percentage (SIMPER) using Bray–Curtis dissimilarities in PAST^50^ (version 4.06) to identify the top 10 taxa contributing to observed differences between overall developmental stage, maternal care groups, and between stage and maternal care groups.

### Taxa overrepresentation and cluster analysis

We used an NBDA from the R package *DESeq2* to identify genera that were significantly overabundant between care groups for each developmental stage (e.g., care: early larvae vs. no-care: early larvae), or between developmental stages only (e.g., early larvae vs. late larvae). This resulted in four comparisons for each stage and care group and six comparisons between stages only; this totaled 20 comparisons. Prior to analysis, *DESeq2* requires that there be at least one variable with no zeros present in any of the samples. Every taxon in our metatranscriptomic data had at least one zero abundance value in at least one sample. To circumvent this, a pseudo taxon with a value of 1 across all 190 samples was added to allow *DESeq2* to properly run and normalize the dataset. Next, the normalized dataset was used for WGCNA. Briefly, missing data or zero-variance data are checked and/or removed; this resulted in the removal of the pseudo taxon. Next, outlier samples were removed if they were more than five standard deviations from other samples via hierarchal clustering of samples; this resulted in the removal of three samples (J11-4, K11-2, and H120-9); the remaining 187 samples we kept for further analysis. Next, analysis was done on developmental stage (e.g., early larvae) or developmental stage + maternal care group (e.g., care: early larvae), which were one-hot coded to convert categorical terms into binary code where 1 indicates a sample is represented by the category and 0 indicating the sample is absent from the category. A range of powers was tested, and a soft-threshold power of 10 was selected, along with a network type set as signed and a minimum module size of 20 (Fig. S6B). All modules that had significant, positive correlations were recorded and analyzed further for hub taxa based on absolute GS ≥ 0.2 and absolute MM ≥ 0.8 using cut-offs obtained from studies^98,99^.

### Random forest classification

The R package *randomForest*^51^ was used for random forest classification to test if gene expression and metagenomic data can clearly predict the developmental stage or maternal care group of offspring. For the metagenomic data, we trained several random forest classifiers (RFCs), which included the complete metatranscriptomic data (i.e., all six domains), only the bacterial domain, only the fungal domain, and a combined bacterial+fungal domain dataset, to predict either overall developmental stage or care group, RFC testing was also done on the normalized gene expression dataset. Prior to RFC on gene expression (20,825 genes), we filtered out DEGs with 15 or fewer counts, shrinking the data to 16,023 genes. The RFCs were trained using 9 training set sizes (10–90%, increasing by 10%), with each training set size run 10 times to obtain an average classification accuracy. This resulted in 90 trials per RFC. Before each RFC, we ran the TUNERF method to determine an optimal mtry value (used to determine the number of features to select for the RFC to minimize error) for 5000 runs (ntree = 5000). The R package *caret*^104^ was used for the confusion matrix, which summarizes the overall performance of the RFC. The R package *randomForestExplainer*^105^ was used to determine the top 10 important features (i.e., taxa or genes) that influenced the RFC model accuracy and was done for each trial run at the 80% training set size. Results summarized and compared between RFCs will focus on 80% of samples withheld.

### TF enrichment

To identify candidate TF binding sites enriched upstream of DEGs, we implemented STREME^42^ from the MEME (Multiple Em for Motif-Elicitation) package (version 5.4.1) to find significantly enriched motifs 5kbp upstream of all unique DEGs for each stage, all upregulated DEGs for each stage comparison, all unique DEGs for each maternal care group, and finally, for all upregulated DEGs for each stage + group using an *E*-value cut-off of 0.05. Significant motifs from STREME were used in subsequent MEME suite packages for motif identification and enrichment. We used the TOMTOM^106^ webserver to identify matched motifs using the JASPAR^107^ non-redundant core 2022 database for vertebrates and insects separately using default settings. Then we implemented GOMO^108^ (Gene Ontology for Candidate Motifs) to assess the functional role of identified significant motifs using default settings.

### Integration of gene expression and metatranscriptomic data

Finally, to detect any patterns or associations between the metagenome and host gene expression, we used the R package *mixOmics*^52,53^ to integrate metagenomic abundance data with gene expression data using sparse partial least squares (sPLS) regression analysis. The sPLS function integrates two datasets measured on the same individuals (e.g., host gene expression and metatranscriptomic data) to detect highly correlated (positive or negative) variables. We filtered the normalized host gene expression data (20,825 genes) to include only DEGs that had a log2foldC > 1. Next, we further filtered these DEGs to focus on hub DEGs from WGCNA that had a GS value > 0.5. This resulted in 32 top genes to be further cross-analyzed with metatranscriptomic data. Next, we filtered the metatranscriptomic genus abundance data to focus only on the top contributors based on SIMPER analyses. This totaled 11 genera. Then we split our filtered gene expression and metatranscriptomic datasets into samples for each developmental stage and group setting (e.g., care; early larvae). We performed sPLS on the filtered datasets for each development and group setting using ncomp = 2 (for two components), mode = regression (to fit a linear relationship between the variables), and near.zero.var = FALSE (since our filtered data is a small portion of the entire dataset and would have fewer zeros throughout). Correlation matrices between genes and taxa were obtained, and only the top 15 positive and negative correlations were extracted for network visualization using Cytoscape^109^ (version 3.9.1).

### Statistics and reproducibility

This study examines transcriptomic and metatranscriptomic sequence data extracted from 190 *C. calcarata* individuals reared with or without maternal presence from early larval to callow stages. For comparisons between maternal presence and absence, in total, 95 individuals were reared with mothers, and 95 individuals were reared without mothers. A sample size of 5 individuals was used for each developmental stage and maternal care group (5 replicates per individual stage and care group), resulting in a total of 40 individuals reared for early larval development, 50 reared for late larval development, 90 reared for pupal development, and 10 reared to the callow stage. Methods for RNA extraction, library preparation, and sequencing, mapping to reference genome, extraction of transcriptomic and metatranscriptomic data, taxonomy classification, and TF enrichment are explained in the methods section.

All statistical analyses were performed in R. Testing the difference in *Aspergillus* abundance between care groups for early larvae, and late larvae was done using a two-tailed t-test, with the *t.test* function in R and var.equal set to TRUE. PERMANOVA for Bray–Curtis dissimilarity beta diversity was performed, followed by ANOVA for beta dispersion and Tukey HSD to determine significantly different stages and care groups. ANOVA for Shannon–Weiner alpha diversity estimates were also performed, followed by post hoc Tukey HSD to identify significantly different groups. In addition, Bartlett’s test was not significant on Shannon–Wiener alpha indices, so the Kruskal–Wallis test was used, followed by post hoc Dunn’s test to determine significantly different groups. Data were deemed significant when *P* values were less than 0.05.

Integration of gene expression and microbiome composition data identified important positive and negative correlations between genes and taxa for individuals reared with and without mothers across the developmental stages, using correlations obtained from the *mixOmics* R package, with details explained in Section “Integration of gene expression and metatranscriptomic data.”

### Reporting summary

Further information on research design is available in the Nature Portfolio Reporting Summary linked to this article.

### Supplementary information

Supplementary Information

Description of Additional Supplementary Files

Supplementary Data 1

Supplementary Data 2-28

Reporting Summary

## Data Availability

Raw reads for RNA sequencing are available on the NCBI Sequence Read Archive under BioProject PRJNA926970. Source data used to generate figures and results in this study are available within the paper in Supplementary Data 1 file.

## Code availability

The code used to obtain results is available upon request.
