# Supplementary material for: The effects of maternal care on the developmental transcriptome and metatranscriptome of a wild bee
Source: Commun Biol. 2023 Sep 14;6:904. doi: 10.1038/s42003-023-05275-2 (PMC10502028; doi:10.1038/s42003-023-05275-2)
Supplement: Supplementary file 1 — Supplementary Information [file 42003_2023_5275_MOESM1_ESM.pdf]

SUPPLEMENTARY FIGURES

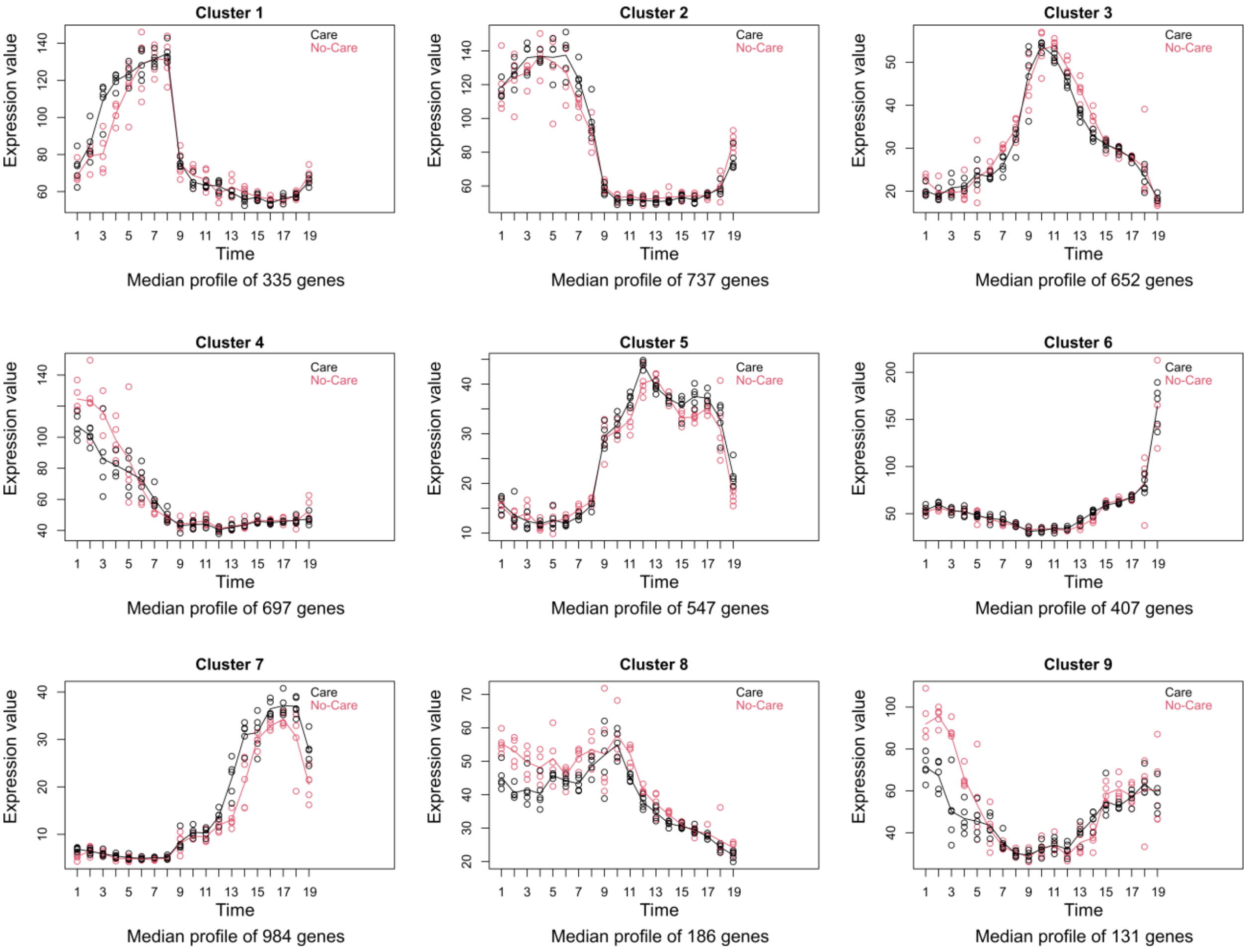

Figure S1. MaSigPro clustering of genes. In total, 9 distinct clusters were found.

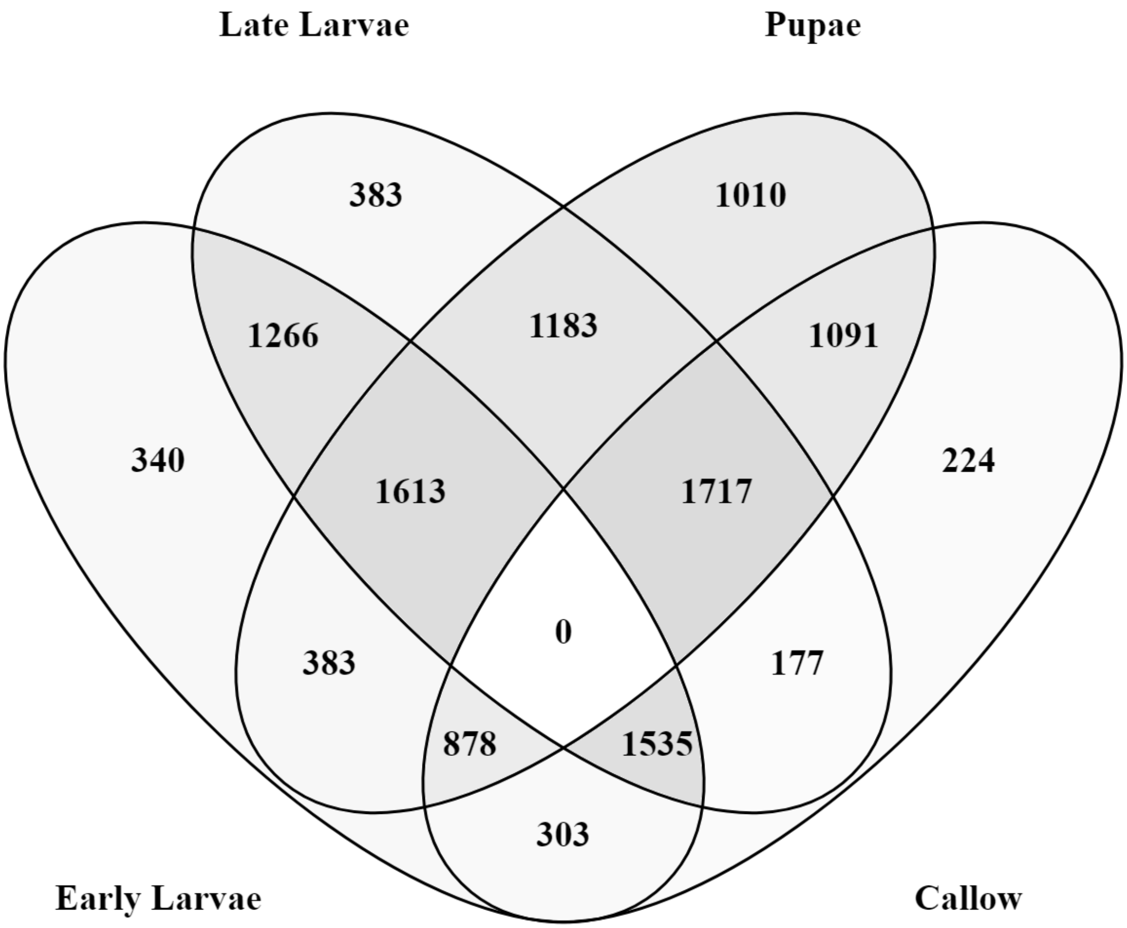

Figure S2. The number of significantly upregulated differentially expressed genes identified across development in *Ceratina calcarata*.

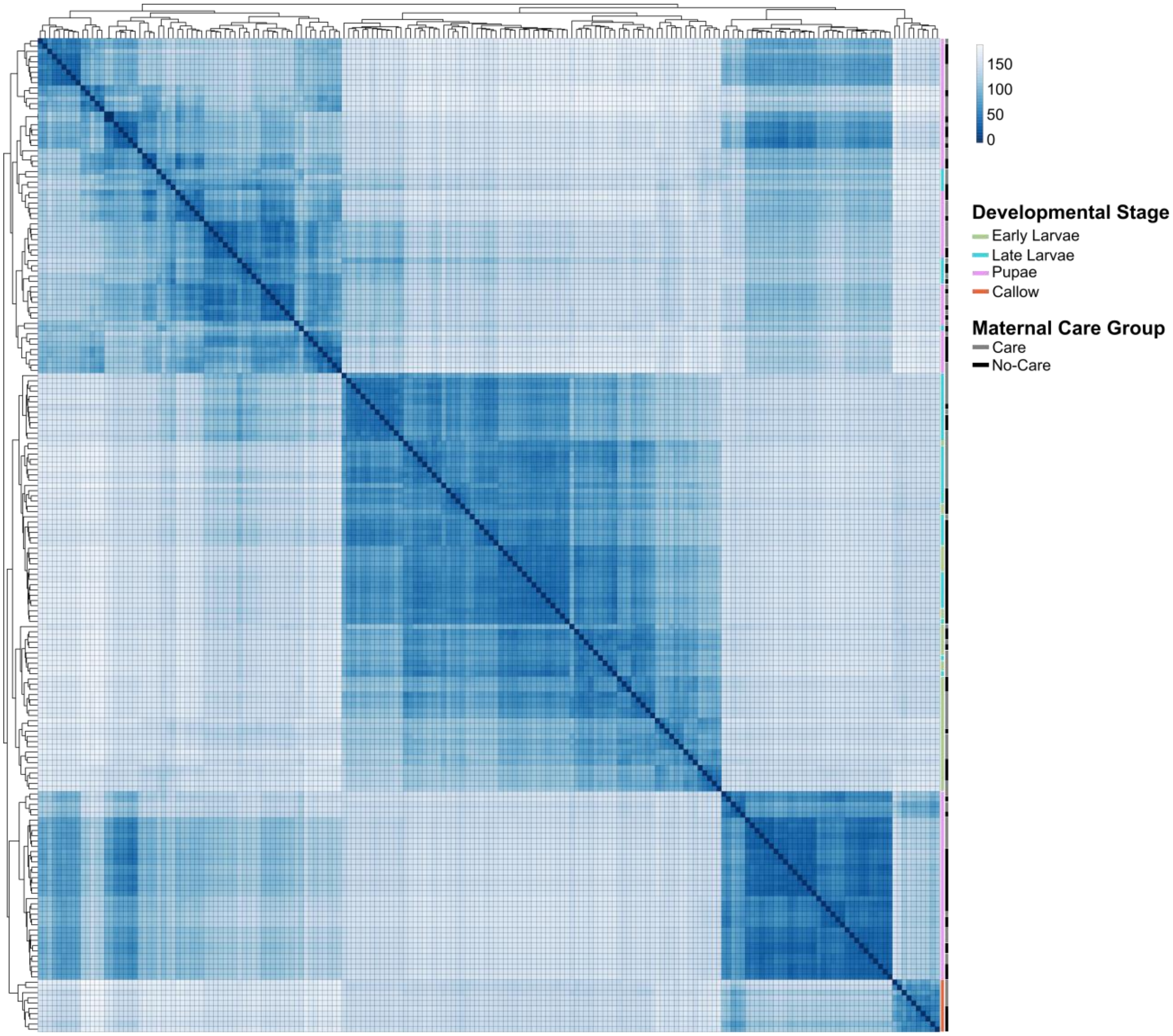

Figure S3. Heatmap of the developmental stages of *C. calcarata* from the early larval to the callow stage in both the presence of maternal care (the care group) and in the absence of maternal care (the no-care group) based on the sample-to-sample distances. The colored columns indicate the developmental stage (first column) and maternal care group (second column).

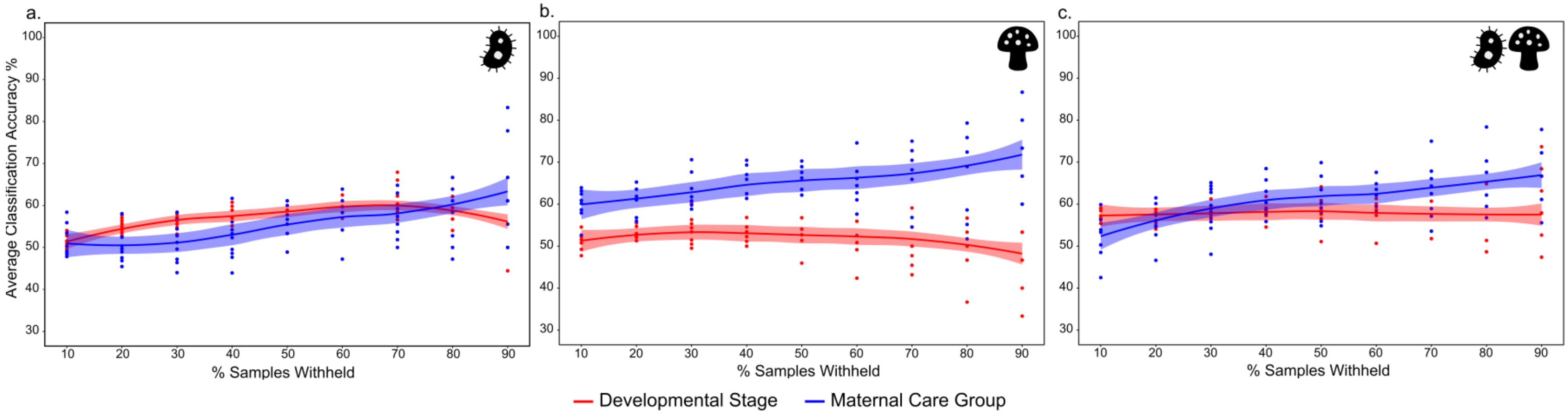

Figure S4. Overall average performance accuracy for each model is shown for separate random forest classifiers (RFC) that test if overall developmental stage or maternal care group can predict samples. RFCs assign samples either to overall developmental stage or maternal care group using genus metatranscriptomic abundance data for (A) bacteria, (B) fungi, or (C) bacteria+fungi. RFC training was done from 10% to 90% of samples withheld. Error bars generated using the loess smoothing curve method. Full results for metatranscriptomic data are shown in **Tables S23** and **S24** for developmental stage and care group, respectively.

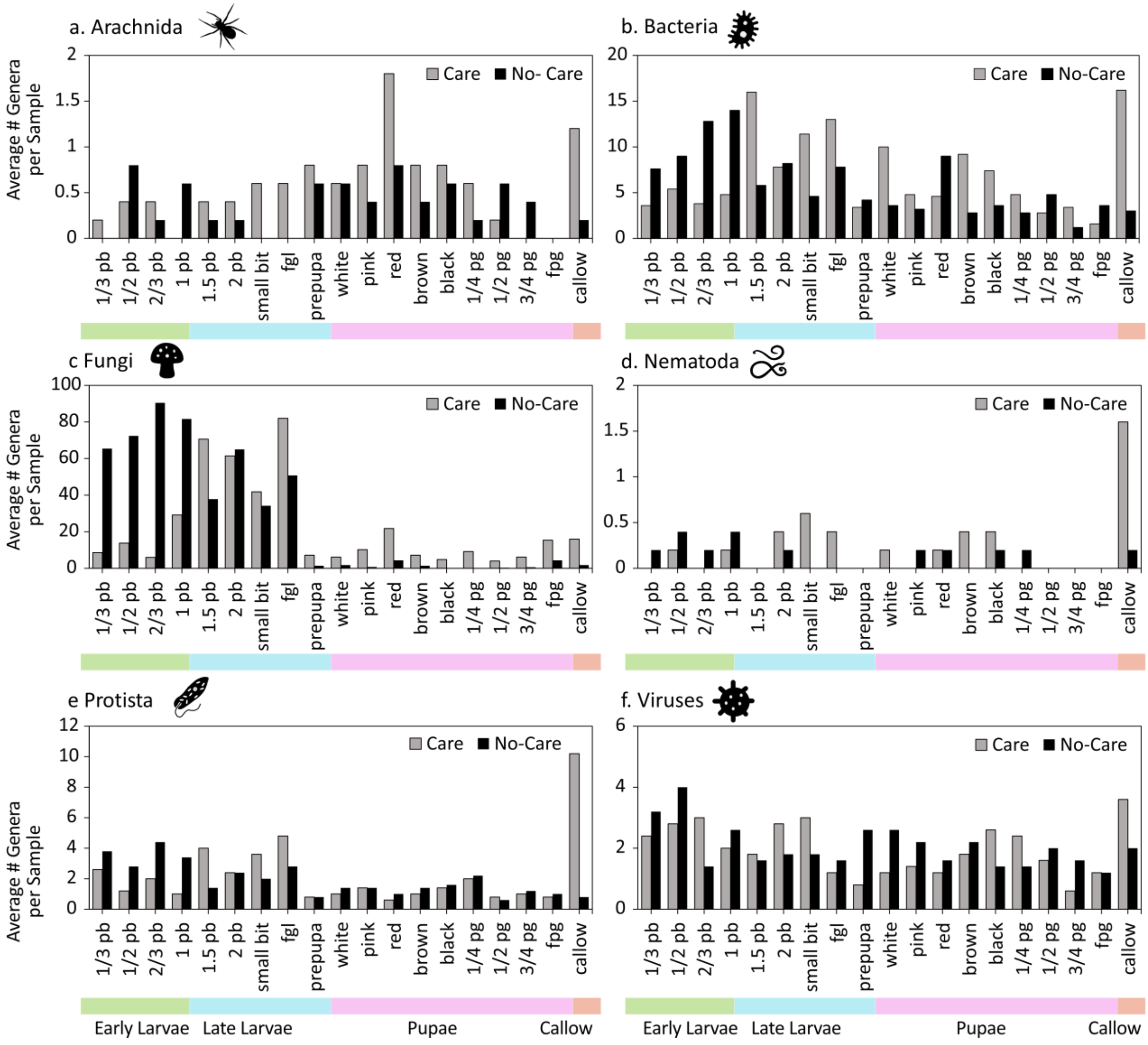

Figure S5. Average number of genera per sample for each domain across individual developmental stages of *Ceratina calcarata* and across different maternal care groups (N = 190 samples).

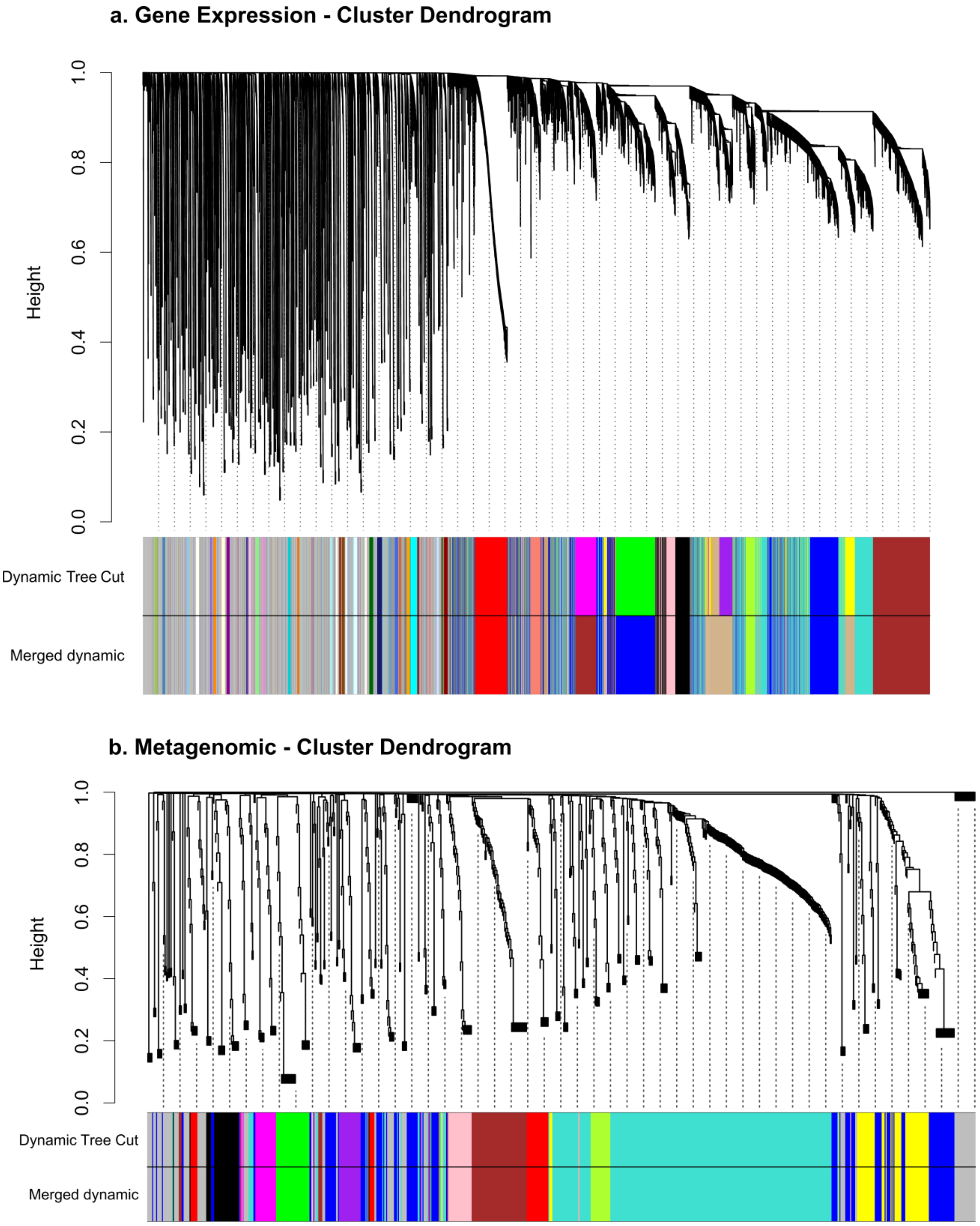

Figure S6. WGCNA module dendrograms for (a) gene expression and (b) metagenomic data using both dynamic tree cut and merged dynamic cut. Gene expression modules are determined

using module size 50, soft threshold power of 6, for an unsigned network type, and metagenomic modules are based on module size 20, soft threshold power of 10, for a signed network type.
