# Supplementary material for: The effects of maternal care on the developmental transcriptome and metatranscriptome of a wild bee
Source: Commun Biol. 2023 Sep 14;6:904. doi: 10.1038/s42003-023-05275-2 (PMC10502028; doi:10.1038/s42003-023-05275-2)
Supplement: Supplementary file 2 — Description of Additional Supplementary Files [file 42003_2023_5275_MOESM2_ESM.pdf]

Description of Additional Supplementary Files

File name: Supplementary Data 1
Description: Source data used to generate the figures and results in this study

File name: Supplementary Data 2
Description: The morphological characterization of the twenty developmental stages of Ceratina calcarata (Adapted from Rehan & Richards, 2010).

File name: Supplementary Data 3
Description: Summary of raw read counts, count of mapped reads, count of unmapped reads, and counts of metaspades contigs before and after contaminant removal.

File name: Supplementary Data 4
Description: Masigpro gene clusters identified. Clusters 4 and 9 represent early larvae development, clusters 1, 2, and 8 for late larvae development, clusters 3 and 5 were combined to represent pupal development, and clusters 6 and 7 for callow (adult) development.

File name: Supplementary Data 5
Description: Differentially expressed genes (DEGs) between overall developmental stages.

File name: Supplementary Data 6
Description: Displaying the significant TopGO terms for all upregulated DEGs (cells A7-AA7) and unqiuely upregulated DEGs (cells AC7-BC7) per developmental stage.

File name: Supplementary Data 7
Description: Differentially expressed genes between care groups.

File name: Supplementary Data 8
Description: Displaying the significant TopGO terms for upregulated differentially expressed genes throughout development in care and no care conditions based on DESeq2 results

File name: Supplementary Data 9
Description: Differentially expressed genes between care and no-care for each overall developmental stage and across all overall developmental stages.

File name: Supplementary Data 10
Description: Displaying the significant TopGO terms for upregulated differentially expressed genes throughout development in care and no care conditions based on DESeq2 results.

File name: Supplementary Data 11
Description: Weighted gene co-expression network analysis (WGCNA) for key modules for maternal care groups (care vs. no care) for each overall developmental stage (early larvae, late larvae, pupal, callow) for all genes.

File name: Supplementary Data 12
Description: Displaying the significant TopGO terms for upregulated differentially expressed genes throughout development based on WGCNA significant modules with hub genes (see Table S10).

File name: Supplementary Data 13
Description: Weighted gene co-expression network analysis (WGCNA) for key modules for maternal care groups (care vs. no care) for each overall developmental stage (early larvae, late larvae, pupal, callow) for all genes.

File name: Supplementary Data 14
Description: Displaying the significant TopGO terms for upregulated differentially expressed genes throughout development and cross maternal care groups based on WGCNA results with significant modules with hub genes (see table S12).

File name: Supplementary Data 15
Description: Summary of BLASTed contig counts for each domain per sample site after removal of contaminants and contigs < 100bp.

File name: Supplementary Data 16
Description: Bray-Curtis dissimilarity and Shannon diversity statistics and significance tests across the four overall developmental stages (early larvae, late larvae, pupal, and callow) for Ceratina calcarata.

File name: Supplementary Data 17
Description: Shannon diversity and Bray-Curtis dissimilarity statistics for maternal care for the four overall developmental stages (early larvae, late larvae, pupal, and callow) for Ceratina calcarata.

File name: Supplementary Data 18
Description: Random forest classifier for maternal care.

File name: Supplementary Data 19
Description: Random forest classifier for developmental stage.

File name: Supplementary Data 20
Description: Genus-level random forest classifier summary for maternal care (care vs. no care).

File name: Supplementary Data 21
Description: Genus-level random forest classifier summary for overall development (early larvae, late larvae, pupal, and callow).

File name: Supplementary Data 22
Description: Top genera for taxa across seven domains extracted from Ceratina calcarata from each major developmental stage (early larvae, late larvae, pupal, callow) contrasted between control and experimental groups.

File name: Supplementary Data 23
Description: SIMPER analysis to identify top genera contributions for overall developmental stages and maternal care using Bray-Curtis dissimilarities across categories.

File name: Supplementary Data 24
Description: Overrepresented genera determined from R package DESeq2 between maternal care groups (care vs. no care) for each overall developmental stage, and between developmental stages.

File name: Supplementary Data 25
Description: Weighted gene co-expression network analysis (WGCNA) for key modules for maternal care groups (care vs. no care) for each overall developmental stage (early larvae, late larvae, pupal, callow) at the genus level.

File name: Supplementary Data 26
Description: Weighted gene co-expression network analysis (WGCNA) for key modules for overall developmental stage (early larvae, late larvae, pupal, callow) across all samples (N=190) at the genus level.

File name: Supplementary Data 27
Description: Significant transcription factor (TF) matches identified by TOMTOM to the enriched motifs (E-value < 0.05) determined from STREME analyses for the significant categories only.

File name: Supplementary Data 28
Description: GO enrichment for enriched transcription factor binding site (TFBS) motifs determined from STREME for each of the three categories: (1) maternal care group, (2) developmental stage, (3) care group + stage.
