# Supplementary material for: The effects of maternal care on the developmental transcriptome and metatranscriptome of a wild bee
Source: Commun Biol. 2023 Sep 14;6:904. doi: 10.1038/s42003-023-05275-2 (PMC10502028; doi:10.1038/s42003-023-05275-2)
Supplement: Supplementary file 5 — Reporting Summary [file 42003_2023_5275_MOESM5_ESM.pdf]

Corresponding author(s): *Double-anonymous peer review submissions: write DAPR and your manuscript number here instead of author names.*

Last updated by author(s): *YYYY-MM-DD*

Reporting Summary

Nature Portfolio wishes to improve the reproducibility of the work that we publish. This form provides structure for consistency and transparency in reporting. For further information on Nature Portfolio policies, see our Editorial Policies and the Editorial Policy Checklist.

Statistics

For all statistical analyses, confirm that the following items are present in the figure legend, table legend, main text, or Methods section.

n/a	Confirmed	
☐	☒	The exact sample size (*n*) for each experimental group/condition, given as a discrete number and unit of measurement
☐	☒	A statement on whether measurements were taken from distinct samples or whether the same sample was measured repeatedly
☐	☒	The statistical test(s) used AND whether they are one- or two-sided<br>*Only common tests should be described solely by name; describe more complex techniques in the Methods section.*
☐	☒	A description of all covariates tested
☐	☒	A description of any assumptions or corrections, such as tests of normality and adjustment for multiple comparisons
☐	☒	A full description of the statistical parameters including central tendency (e.g. means) or other basic estimates (e.g. regression coefficient) AND variation (e.g. standard deviation) or associated estimates of uncertainty (e.g. confidence intervals)
☒	☐	For null hypothesis testing, the test statistic (e.g. *F*, *t*, *r*) with confidence intervals, effect sizes, degrees of freedom and *P* value noted<br>*Give P values as exact values whenever suitable.*
☒	☐	For Bayesian analysis, information on the choice of priors and Markov chain Monte Carlo settings
☒	☐	For hierarchical and complex designs, identification of the appropriate level for tests and full reporting of outcomes
☒	☐	Estimates of effect sizes (e.g. Cohen's *d*, Pearson's *r*), indicating how they were calculated

Our web collection on statistics for biologists contains articles on many of the points above.

Software and code

Policy information about availability of computer code

Data collection	Data used in this study were manually obtained through field collection, lab preparation, sequencing (performed by Genome Quebec). The reference Ceratina calcarata genome was obtained from NCBI Genbank using bioproject ID PRJNA791561.
Data analysis	We used and cited publicly available programs to analyze data for this study, including STAR, BLASTn, metaSPADES, PAST v 4.06, Entrez Direct E-Utilities (efetch version 15.3), and STREME (from MEME package). We also used the MEME webserver to perform TOMTOM and GOMO analysis along with JASPAR nr-core 2022 databases for vertebrates and insects. We used R script packages DESeq2, TopGO, MaSigPro, WGCNA, randomForest, randomForestExplainer, vegan, mixOmics.

For manuscripts utilizing custom algorithms or software that are central to the research but not yet described in published literature, software must be made available to editors and reviewers. We strongly encourage code deposition in a community repository (e.g. GitHub). See the Nature Portfolio guidelines for submitting code & software for further information.

Data

Policy information about availability of data

All manuscripts must include a data availability statement. This statement should provide the following information, where applicable:

- Accession codes, unique identifiers, or web links for publicly available datasets
- A description of any restrictions on data availability
- For clinical datasets or third party data, please ensure that the statement adheres to our policy

All newly generated transcriptome and metatranscriptome data used in this study can be accessed through NCBI Sequence Read Archive under BioProject PRJNA926970

Research involving human participants, their data, or biological material

Policy information about studies with human participants or human data. See also policy information about sex, gender (identity/presentation), and sexual orientation and race, ethnicity and racism.

Reporting on sex and gender	N/A
Reporting on race, ethnicity, or other socially relevant groupings	N/A
Population characteristics	N/A
Recruitment	N/A
Ethics oversight	N/A

Note that full information on the approval of the study protocol must also be provided in the manuscript.

Field-specific reporting

Please select the one below that is the best fit for your research. If you are not sure, read the appropriate sections before making your selection.

☐ Life sciences ☐ Behavioural & social sciences ☒ Ecological, evolutionary & environmental sciences

For a reference copy of the document with all sections, see nature.com/documents/nr-reporting-summary-flat.pdf

Ecological, evolutionary & environmental sciences study design

All studies must disclose on these points even when the disclosure is negative.

Study description	Assessment of a wild bee (Ceratina calcarata) transcriptomic data to elucidate the effects of maternal care on the developmental transcriptome (from larval to adult) and maternal care effects on the metatranscriptome across development.
Research sample	We obtained transcriptomic data from 190 Ceratina calcarata individuals across various developmental stages (early larvae, late larvae, pupae, and callow) and from different maternal care groups (mothers present; reared naturally in the field or mothers absent; reared in the lab without mothers) to allow for calculation of average taxonomic abundance and gene expression patterns.
Sampling strategy	Ceratina calcarata are a facultatively social wild bee in North America, and are an excellent model species to observe the influence of maternal care both on gene expression patterns through development and the change in microbiome community through development. Each group (stage and care) is represented by at least N=5 individuals (care:callows and no-care: callows, N=5 each) through N=45 individuals (care:pupae and no-care:pupae, N=45 each).
Data collection	Ceratina calcarata nests were collected from sumac and raspberry stems in Toronto, Canada."Care" individuals were individuals already raised by mothers in the field to a certain developmental stage. "No-care" individuals were reared in lab to various developmental stages in the absence of mothers. RNA extraction was done on all 190 individuals and sequencing conducted at Genome Quebec. Raw reads were further processed at Genome Quebec and transcriptomic and metatranscriptomic analysis was performed by KDC, MS, JH, and SMR.
Timing and spatial scale	Samples were collected in Toronto, Canada June-July 2019.
Data exclusions	No data were excluded from our analyses.
Reproducibility	Analysis of transcriptomic and metatranscriptomic data were re-run to confirm consistency of results.
Randomization	Gene expression and taxonomic abundance were analyzed in various "groups" including (1) all care vs. no-care samples, (2) early

Randomization	larvae, late larvae, pupae, vs. callows, and (3) stage+group which includes care:early larvae, no-care:early larvae, care:late larvae, no-care:late larvae, care: pupae, no-care: pupae, and care:callow and no-care:callow. We then compared our findings to previous studies on bee maternal care, development, and microbiome data.
Blinding	Blinding was not necessary nor possible for our study. Our findings are based on the results of rigorous analysis on gene expression and microbiome community across development and maternal care groups. Awareness of sample type and groupings was necessary for comparative data analysis in our study.

Did the study involve field work? ☒ Yes ☐ No

Field work, collection and transport

Field conditions	Samples were collected on York University, Keele Campus in Toronto, Canada. All stems were collected from forest margins and semi-shaded environments.
Location	Field collections were done in Toronto, Ontario, Canada (43.7735 N, 79.5019 W).
Access & import/export	Sample locations were from public university property allowing free access to extract nests. No permits were required.
Disturbance	Samples were collected by extracting nests from sumac and raspberry stems. 143 nests in total were obtained with minimal disturbance to surrounding plants and habitat.

Reporting for specific materials, systems and methods

We require information from authors about some types of materials, experimental systems and methods used in many studies. Here, indicate whether each material, system or method listed is relevant to your study. If you are not sure if a list item applies to your research, read the appropriate section before selecting a response.

Materials & experimental systems

n/a	Involved in the study	
☒	☐	Antibodies
☒	☐	Eukaryotic cell lines
☒	☐	Palaeontology and archaeology
☐	☒	Animals and other organisms
☒	☐	Clinical data
☒	☐	Dual use research of concern
☒	☐	Plants

Methods

n/a	Involved in the study	
☒	☐	ChIP-seq
☒	☐	Flow cytometry
☒	☐	MRI-based neuroimaging

Animals and other research organisms

Policy information about studies involving animals; ARRIVE guidelines recommended for reporting animal research, and Sex and Gender in Research

Laboratory animals	This study invovled animals reared in a laboratory.
Wild animals	This study involved the live capture of wild animals (Ceratina calcarata) at various developmental stages.
Reporting on sex	Ceratina calcarata were sexed only after the pupal stage where sexing is viable. All pupae and callows were identified as female. Sexing of larvae was not required. Sexing of Ceratina calcarata individuals was not required for our study design.
Field-collected samples	No-care offspring were reared in a laboratory at 23 degrees Celsius and 50% relative humidity. Field collected individuals were flash frozen in liquid nitrogen and stored in a -80 degrees Celsius freezer.
Ethics oversight	The bees used in our study are not identified as at-risk species in North America nor under any extinction concern. No ethical approval or guidance was needed for our study.

Note that full information on the approval of the study protocol must also be provided in the manuscript.
